# A Best Practices Case Study for Scientific Collaboration between Researchers and Managers

**DOI:** 10.1002/fsh.10536

**Published:** 2020-12-19

**Authors:** Tammy J. Newcomb, Paul W. Simonin, Felix A. Martinez, W. Lindsay Chadderton, Jon M. Bossenbroek, Becky Cudmore, Michael H. Hoff, Reuben P. Keller, Berkley D. Ridenhour, John D. Rothlisberger, Edward S. Rutherford, Scott Van Egeren, David M. Lodge

**Affiliations:** ^1^ Michigan Department of Natural Resources Lansing MI; ^2^ Cornell University Department of Ecology and Evolutionary Biology Ithaca NY; ^3^ National Oceanic and Atmospheric Administration, National Centers for Coastal Ocean Science Ann Arbor MI; ^4^ The Nature Conservancy, Great Lakes Project South Bend IN; ^5^ Department of Environmental Sciences and Lake Erie Center University of Toledo Toledo OH; ^6^ Fisheries and Oceans Canada Burlington ON Canada; ^7^ U.S. Fish and Wildlife Service (retired), Fish and Aquatic Conservation Bloomington MN; ^8^ Loyola University Chicago Institute of Environmental Sustainability Chicago IL; ^9^ The Nature Conservancy Moscow ID; ^10^ USDA Forest Service Washington D.C.; ^11^ National Oceanic and Atmospheric Administration Great Lakes Environmental Research Laboratory Ann Arbor MI; ^12^ Wisconsin Department of Natural Resources Rhinelander WI; ^13^ Cornell University Cornell Atkinson Center for Sustainability Ithaca NY; ^14^ University of Notre Dame Environmental Change Initiative South Bend IN (former)

## Abstract

Effective engagement among scientists, government agency staff, and policymakers is necessary for solving fisheries challenges, but remains challenging for a variety of reasons. We present seven practices learned from a collaborative project focused on invasive species in the Great Lakes region (USA‐CAN). These practices were based on a researcher–manager model composed of a research team, a management advisory board, and a bridging organization. We suggest this type of system functions well when (1) the management advisory board is provided compelling rationale for engagement; (2) the process uses key individuals as communicators; (3) the research team thoughtfully selects organizations and individuals involved; (4) the funding entity provides logistical support and allows for (5) a flexible structure that prioritizes management needs; (6) a bridging organization sustains communication between in‐person meetings; and (7) the project team determines and enacts a project endpoint. We predict these approaches apply equally effectively to other challenges at the research–management–policy interface, including reductions of water pollution, transitions to renewable energy, increasing food security, and addressing climate change.

**Figure:**
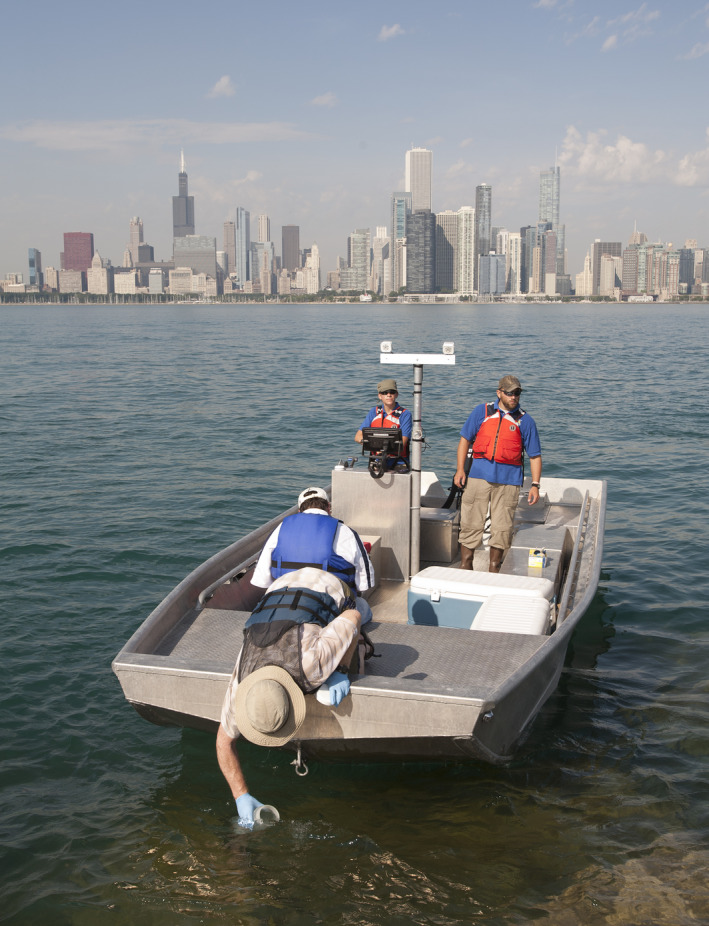
Colleagues from the University of Notre Dame (research team members), The Nature Conservancy (bridging organization), and the U.S. Fish and Wildlife Service collecting eDNA water samples for Asian carp surveillance along the Chicago waterfront. Photo credit: Paul Merideth

## INTRODUCTION

Tension is often present between scientists and managers in natural resource management. At its worst, this tension can be expensive, polarizing, and unproductive. Examples of this tension include scientists broadly publicizing research findings before managers have an opportunity to understand the research and its management implications, or managers ignoring research results when implementing management actions. Consequently, the public may demand an immediate management response without understanding the uncertainties in the research conclusions or the uncertainties of the efficacy of any management interventions. Managers may therefore resist change in management in the face of the risk associated with both uncertainties. In a recent project, we embraced the challenge that this tension poses between research and management programs, and deliberately developed and engaged in a process that leveraged the energy behind the tension to produce better invasive species management.

Translation of research results into products useful for natural resource managers and policymakers is a goal of many funding programs, and in response, resources are increasingly dedicated to transdisciplinary research likely to accomplish that goal (Klenk et al. 2015). Since 1997, the National Science Foundation has evaluated proposals based on their “broader impacts” in addition to their “intellectual merit” (National Science Board 2005). The Canadian Science Advisory Secretariat Process brings together practitioners and scientists to develop research questions (available: https://bit.ly/30jNAZN). Similarly, the National Oceanic and Atmospheric Administration (NOAA) National Centers for Coastal Ocean Science (NCCOS) Competitive Research Program integrates research and management, and suggests several models that their funded programs may implement to assist in the transfer of research results to policymakers (Turner et al. 2000; Bosch et al. 2003; Liu et al. 2008). This push for transferring research outcomes to management application has produced a variety of engagement models (e.g., Liu et al. 2008; Wittmann et al. 2015; Braun et al. 2016), including adaptive management (Walters 1986), structured decision making (Gregory et al. 2012), and a Knowledge Action Framework (Nguyen et al. 2016). These and other models aim to facilitate knowledge exchange and interconnectedness among research, management, and policy communities so decisions surrounding a specific project or issue are informed by new information (Bornmann 2014; Enquist et al. 2017; Wall et al. 2017), but the resources required to implement adaptive management or structured decision making, for example, can be daunting for natural resource agencies (Walters 2007).

Other strategies to facilitate research transfer range from governance models to generalized practices. In one governance model, the Great Lakes Fishery Commission implements a basin‐wide research program with tacit, coordinated interactions to gain management information needs that will help to guide research investment (e.g., GLFC 2007). In less formal strategies, the focus is on ensuring interaction between researchers and managers as individual research projects are planned and conducted (e.g., National Oceanic and Atmospheric Administration’s National Center for Coastal Ocean Science, Competitive Research Funding [NOAA NCCOS CRP]). State and federal natural resource agencies may have in‐house research organizations that work closely with agency resource managers to provide research relevant to agency decisions and activities (e.g., the Research Section of the Michigan Department of Natural Resources Fishery Division [available: https://bit.ly/36fNczf]; U.S. Forest Service’s Research and Development branch; Ryan 2003). In other cases, the management community is integrated into the research program after funding is awarded (e.g., some National Science Foundation grants).

Increasingly, such efforts span institutions because resources for government staff and travel are declining, and scientific expertise is being outsourced to other government agencies, academic institutions, independent scientific consultants, or NGOs. Simultaneously, demands by funding organizations for efficiency and accountability are increasing. Reliance on scientific expertise external to government has thus increased, and brings with it increased challenges, three of which we summarize below.

### Common Challenges or Tensions at the Research–Management–Policy Interface

#### Differing Timelines for Products and Results

Scientists (academic, NGO, and agency) typically have longer project time frames than desired by managers (those with jurisdictional and regulatory authority) and policymakers, making it sometimes difficult for scientific research to inform real‐time management decisions. A typical research cycle takes several years to obtain funding, implement the research, and distribute findings through a publication process. Scientists consider a research question to be answered only after it has been peer‐reviewed and published and are sometimes resistant to release information or data in advance of peer review as a matter of scientific rigor. Managers and policymakers, however, are often asked to respond quickly and may be bound or directed by time scales informed biologically by organism reproduction cycles or harvest seasons or sociopolitical influences related to news cycles, budgetary fluctuations, and even elections.

#### Lack of Alignment in Interests between Research and Management

The practical needs of managers are often poorly aligned with the research goals and outputs expected of scientists. Professional reward systems enhance this disconnect by encouraging academics to conduct broad‐scale studies, which are more likely to be published in widely read prestigious journals. Conversely, managers and policymakers can have important, geographically specific problems at smaller scales (e.g., individual watersheds, lakes, or management zones), especially at the state level where many environmental decisions are made. Legislative action, political pressure, or agency leadership may require expedient enactment of programs or rules, despite incomplete information.

#### Uncertainty as a Hurdle to Acceptance, Interpretation, and Application

Uncertainty in scientific results can prevent acceptance or adoption of research outputs if: (1) the implication of the results or uncertainty around those results is not well understood by managers, or (2) the process by which data are analyzed and interpreted is difficult for managers to understand. When a researcher communicates uncertainty in results, policymakers may mistakenly conclude that the results are unreliable, when in fact adoption of the results would improve management. Such hurdles can arise if managers were not involved in the formation of research questions or their concerns and input were not understood by the research team. Additionally, although researchers and managers commonly discuss uncertainty inherent in the results of any scientific study, the public may have limited understanding of the issues. Because managers are held accountable for their decisions by the public, scientific uncertainty can contribute to management hesitancy, especially when the status quo is a politically safer option. This hesitancy can become paralyzing in practice if actions could result in hardship for certain stakeholders and thus greater public controversy (Shackley and Wynne 1996).

The goal of this paper is to provide a model of a collaborative engagement process that helped us overcome the potential for management paralysis, and was effective in developing research products that informed management and policy in a timely fashion. Additionally, best practices are outlined that were informed by this engagement model.

## MODEL FOR COLLABORATIVE ENGAGEMENT

**Figure 1:**
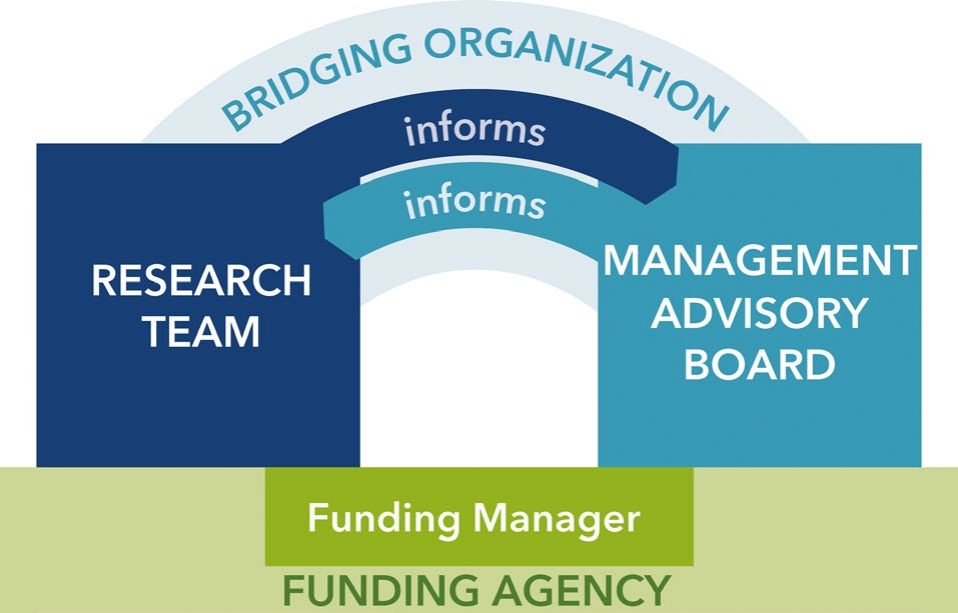
Model for research–management collaboration. The research team and management advisory board play the central role, founded upon resources supplied by a funding agency. Interactions between project components are facilitated by a bridging organization. The work of this bridging organization is crucial to the success of the overall project.

The model that we developed and applied is composed of four main components (Figure 1) that support co‐production of research projects, products, and outcomes by researchers and agency staff. Large‐scale collaborations may find the model particularly applicable, but it may also be informative to smaller project teams. A funding agency, represented by a funding manager, forms the foundation and motivates synergy among the three other components by providing resources to study a topic of research and management interest. A research team and a management advisory board (MAB) form the two pillars of the model and the core components of the overall project team (Figure 1). Finally, a bridging or boundary spanning organization (*sensu* Safford et al. 2017), serves as a member of both the research team and MAB to facilitate communication between these groups.

The research team includes individuals involved in conducting the project research and may be composed of university scientists, government, and non‐profit sector scientists. Ideally, the research team has reached out to potential users of the research outcomes to scope an appropriate proposal. A management advisory board, consisting of potential end users of the research results, is a group established to help guide the research team toward research questions and outputs that are immediately relevant (Figure 1). The MAB should be large enough to provide input from the full range of potential users, but small enough (fewer than 25) to function effectively, and membership should be carefully selected to support the desired knowledge exchange (Hegger et al. 2012). While MAB members’ salaries are unlikely to be supported by the project, the provision of funding for travel to meetings and site visits can be extremely important to retain engagement of agency staff that may be under travel restrictions due to limited budgets. Selection of the MAB may require a formal solicitation letter to agency leadership requesting participation by agency staff and articulating time commitments and expectations to encourage sustained engagement and support for the eventual MAB member(s).

The responsibility of the MAB is to advise the research team on management priorities and information needs, and on how to present research results for optimal effectiveness and application. The MAB is not involved in conducting the research, but has an evolving responsibility during different project phases. During the initiation of the project (post‐funding), the MAB will meet with project scientists to review the project scope, identify avenues of inquiry that have potential for management applications, and study topics of management interest. As the project advances, the MAB tracks research progress, offers suggestions on how to further focus research activities to best meet management needs, and examines preliminary lab or model results that appear inconsistent with agency experiences in the field. The MAB should also identify potential resistance to application of research results and advise how to increase the relevance and applicability of research products. Finally, the MAB will facilitate communication with key agency leadership and/or institutional channels (i.e. agency workgroups or management teams) to enhance transfer of the research to management and policy. Despite the high level of interaction with the research team, the research team remains responsible for the integrity of the scientific process.

The bridging organization exists as a member of both the research team and MAB and facilitates interactions between these groups. Characteristically, the bridging organization should include staff members who are familiar with the research topic, skilled in meeting facilitation, experienced in communicating with large groups, and trusted by both researchers and managers to be an objective party (i.e., analogous to the backbone organization in Braun et al. 2016 and the third party science neutral group in Mac and Palmer 2020). The bridging organization serves as the primary point of contact for project‐related communications, including meeting information (agenda, anticipated outcomes, development of read‐aheads and pre‐work that needs to occur), project publications, or the hosting of webinars to present results or to seek input between group meetings. The bridging organization serves as host for the team meetings by putting thoughtful consideration into meeting logistics that support productive interactions and thus encourage future participation. Logistics include a convenient meeting location in proximity to the participants, pleasant meeting space, catering, interesting fieldtrip opportunities, group dinners, and technology that allows remote participation. These efforts support and develop relationships between the research team and MAB, increase satisfaction and effectiveness of the knowledge exchange (Fazey et al. 2012), and overcome the lack of personal interactions often cited as a primary constraint to research uptake (Schwartz et al. 2017). It is important for the project budget to include funding that allows the bridging organization to facilitate travel to meetings by providing reimbursement or direct payment of participants’ travel expenses when their agency is unable to do so.

## CASE STUDY

This model was applied during our recent project on invasive species risk assessment, detection, and impact forecasting in the Laurentian Great Lakes region, led by the University of Notre Dame and funded by NOAA NCCOS CRP and the U.S. Environmental Protection Agency Great Lakes Restoration Initiative. For this project, a multi‐disciplinary, multi‐institutional (see author affiliations) research team of academic, government, and independent scientists focused on four research topics that were supported and informed by a MAB. The topics included: (1) forecasting spread (for new and existing populations) and bioeconomic impacts of aquatic invasive species in the Great Lakes; (2) environmental DNA surveillance and applied early detection; (3) invasive species surveillance of the bait trade; and (4) risk assessment of invasions from trade in live aquatic organisms. Explicit attention was given to scientific and management uncertainty on all these topics. The MAB was comprised of representatives from state, provincial, and federal government agencies that work in the Great Lakes region.

Membership of the MAB associated with the project was limited to agency representatives to foster open dialogue among the jurisdictional authorities. The engagement of only governmental stakeholders respected the sensitivities of discussing program weaknesses and political pressures and allowed these discussions to occur in an environment of peers. Individual members of the MAB represented a range of management responsibilities including policy advisors, program managers, and biologists from environmental quality and natural resources agencies. To solicit members, participation was requested through informal communications, sent by the lead investigator, to agency staff describing the projects, potential outcomes, a description of the level of engagement (e.g. time, review of materials, meeting participation), and resources available to support travel and meeting participation. The commitments requested of these members were to attend meetings and participate in dialogue, help identify appropriate research questions, help make connections to data sources, critically evaluate project results and directions, provide input on specific requests from the research programs, and report results back to their parent agency staff as the findings emerged.

To be an effective partner with the MAB, the research team maintained the objective stance appropriate to science; without advocating for particular management or policy outcomes, but rather advocating only for the appropriate use of research in evaluating potential policies or management actions. In interactions with the MAB, the research team focused on practical aspects of their work, and not only the novelty or intellectual challenge of the research. The research team embraced the responsibility to clearly communicate their work to the MAB, and to help overcome communication challenges that arose among meeting participants.

Three staff members from The Nature Conservancy (TNC), an NGO with a partnership arrangement with the University of Notre Dame, served as the bridging organization between the research team and the MAB. The skillsets of these staff included leadership and experience in research and management with aquatic invasive species, including previous work with many of the MAB agencies. Staff from TNC were actively engaged on some of the research projects as co‐principal investigators, helped facilitate and set the agenda for meetings, and assisted with project administration, communications, and logistics. Staff from TNC were viewed as trusted partners that effectively leveraged and increased the collaborative spirit that already existed among researchers, managers, and policymakers. As part of the process, the bridging organization often provided check‐ins on the process between the MAB and researchers to provide feedback on any adjustments to meeting needs and communication products and resolve any potential conflicts. For example, early in the process, the MAB requested brief read‐ahead materials from the research team to reduce the amount of information overload at the meetings and facilitate deeper discussions.

Relationships among project participants were maintained through strategic communication throughout the project. Funds for travel and lodging were provided to MAB members to attend in‐person meetings, or telecommunications were made available if they couldn’t attend, to encourage maximum and sustained participation. The research team’s travel was incorporated in their research project budgets. In‐person MAB meetings occurred annually and immediately followed the research teams’ meetings. MAB meetings consisted of brief presentations by research team members followed by equal time for discussion. Meetings included lead personnel from the scientific teams, and at least 1 hour of each meeting was reserved for discussions among MAB agency members only to foster constructive and critical dialogue about the ongoing work. To improve communication between the research teams and the MAB, the research team created a living two‐page document for each sub‐project that reported a summary of the project description, goals, status of research and outreach efforts, a list of outstanding issues needing feedback from the MAB, and the primary research team contact. Materials for each meeting were circulated in advance to allow participant review, with decision points clearly articulated before and during meetings. Informal gathering time was scheduled to strengthen connections among MAB members and the research team. This high level of organization was provided to the team by the bridging organization’s project coordinator and the lead university partners. The development of the relationships through these efforts supported an open, transparent, and engaging dialogue among all participants. Crucial conversations were held in this trusted environment that bolstered understanding and heightened the value of the research outcomes to the management agencies.

### Mutual Benefit to MAB and Research Team

Both the MAB and research teams benefited from the organization and roles described above. Throughout the project, the scientists worked to identify topics within the scope of the funded projects for which interesting intellectual challenges overlapped with high priority management or policy needs. The research team’s pursuit of this convergence between scientific inquiry and practical application—often referred to as Pasteur’s Quadrant (Stokes 2011) or use‐inspired research (Wall et al. 2017)—led to fruitful research and engagement regarding topics such as tools for early invasive species detection, habitat suitability for invasive species, identification of high‐risk invasive species, ship de‐ballasting locations to minimize probability of species spread, risk analysis of invasive species impact, and bioeconomic analysis of management options (Table 1).

**Table 1:** Examples of outcomes and benefits as identified through an informal survey of the research team and management advisory board from their respective participation in the project described herein.

Participant	Outcomes	Examples of outcomes
**Researchers**	New research topic: early detection of invasive species	Prompted and guided exploration of environmental DNA detection techniques. (Jerde et al. 2011, 2013; Nathan et al. 2015; Tucker et al. 2016; Deiner et al. 2017)
	New research topic: trait‐based identification of high‐risk invasive species	Guided by managers toward which taxonomic groups were highest priorities, and which stage of invasion was most critical to assess (Gantz et al. 2015; Drake 2015; Howeth et al. 2016; Lodge et al. 2016; Kramer et al. 2017; Wittmann et al. 2017)
	New research topic: ship deballasting techniques to minimize species spread	Government agencies provided guidance that directed researchers’ work on dispersal of species by ships or currents (Sieracki et al. 2014; Beletsky et al. 2017)
	New research topic: risk analysis of aquatic invasive species bioeconomic impacts and management options	Guidance determined which invasive species vectors, species, and management strategies were chosen for focus of bioeconomic analysis of aquatic invasive species impacts (Zhang et al. 2016)
**Managers**	Strengthening of ongoing efforts	Improved coordination and implementation, e.g., improvements to Ohio’s Asian carp control plan and improvements in Michigan’s and Wisconsin’s ongoing management
	Creation of new regulations and policy	Ohio and Wisconsin’s invasive species tactical plans, Michigan and Wisconsin organisms in trade policies, interstate surveillance plans
	Skill in expert elicitation	U.S. Army Corps of Engineers applied lessons learned from the elicitation process in the Great Lakes Mississippi River Interbasin Separation Study–Brandon Road report
	Professional development and networking	Partnerships fostered between state managers, and between state managers and researchers in a way not possible through scientific conferences
	Resolution of funding challenges and more efficient use of resources	Awareness of research happening in the basin was enhanced, allowing U.S. Environmental Protection Agency Great Lakes Restoration Initiative funding to be more efficiently allocated

For the managers, MAB participation helped inform ongoing management and policy efforts (Table 1). For example, the work of the Council of Great Lakes and St. Lawrence Governors’ and Premiers’ Aquatic Invasive Species Task Force was assisted by the ongoing dialogue and research related to risk assessment. A summary of the risk assessment process developed during the project also informed ongoing live trade regulatory efforts and helped managers respond to inquiries from industry. Thus, in multiple ways, the ongoing work of MAB members was enhanced by their participation on the board (Table 1).

## BEST PRACTICES FOR DEVELOPING A COLLABORATIVE RESEARCH TO MANAGEMENT AND POLICY PROCESS

In developing the best practices outlined below, members of the MAB and research team reflected on lessons learned while enacting the above model as well as our previous experiences with collaborative efforts to transfer research to management. Specifically, why did this process work for us and why was it effective to move the needle on invasive species issues? From those conversations, the following list of best practices was derived that we believe will have broad applicability for increasing the effectiveness of research‐management engagement on many topics of natural resource management and policy. Specifically, we recommend the following seven practices.

### Develop a Compelling Rationale for Engagement with Resource Agencies

The rationale for collaboration between research organizations and management and policy organizations should be well‐defined and compelling, with a shared vision and common agenda or goals (Braun et al. 2016). This rationale will be the “hook” to encourage agency staff to prioritize their participation given competing demands for their time. The rationale for the group’s existence must be identified early and communicated clearly.

### Identify Key Communicators and Their Roles

As the process begins and the community of participants develops, “key translators” are needed to enhance communication between the MAB and the research team. Key translators are those that are excellent cross‐boundary communicators and embrace this role (Enquist et al. 2017). They are keen on seeking to understand the issues of contention and are often sought out by both scientists and MAB members to seek solutions when issues arise throughout the collaborative process. Members of the bridging organization can serve in this role, but one or more members of the research team and MAB may also serve this role. Differences of opinions and direct conversations often lead to a richer understanding of the issue. To inform policy and management through a MAB, the researcher gains a fuller understanding of the range of inputs considered by decision makers, thus informing researchers on how to communicate to scientific output into that agency context. Conversely, managers develop a deeper understanding of the scientific and analytical principles used in the research process. Identifying and facilitating the growth of representatives from the research team—who can provide clear explanation of research activities, products, and goals—is invaluable for transitioning research outputs to management outcomes. The final link is for scientists and managers to use what they learn from one another to advance knowledge and increase the relevance of scientific products and to improve the effectiveness of policies, regulations, and management activities.

Throughout the process, there is a need for listening skills from everyone. Decision makers must engage in dialogue with researchers to have their interests and needs represented. This helps researchers focus on the management imperatives. For example, when most managers agree on a particular point, the scientists get a sense of the relative importance of that issue.

### Carefully Select the Composition of the Management Advisory Board

The MAB should include an appropriate suite of participants relevant to the research topic (though it may be advantageous to limit this to a particular sector, such as government), but be small enough for all members to provide input. After MAB members are identified, an official invitation for their participation should be extended, including an explanation of the rationale and desired outcomes of the project and an estimate of the time commitment involved, ideally outlining the approximate number of meetings, webinars, calls, and document reviews expected. Consideration should be given to both how and to whom this invitation will be made. In many cases, it is desirable to send the official invitation to the prospective MAB member’s supervisor or agency administrator to gain awareness and generate support for the agency’s involvement from agency leaders.

The MAB could include representatives of any organizations that are responsible for managing the resources in question. Participants need to trust each other and their organizations to respect the confidentiality of the MAB discussions. Including a representative from the funding agency on the MAB can facilitate these conversations to avoid the principal investigator (i.e., grant recipient) becoming engaged in mediating between the MAB and the funding agency. Finally, care should be taken to select board members whose interpersonal relationships will be marked by respect and trust, and provide membership with diversity in background and experience.

### Provide Logistical Support for Scientists and MAB

For a large collaborative project and process to function efficiently and within a defined timeline, effective logistical support from the bridging organization is necessary. As a matter of project proposal budgeting, we highly recommend requesting resources for staff time or additional personnel to provide this support through the bridging organization. Communication and updates must be shared in a timely and effective manner, finances must be managed, travel by participants reimbursed, and meetings supported. These activities are most likely to happen through specific staff who are hired or assigned to perform this function, rather than expecting a researcher or manager to take on extensive logistical and coordination tasks as ancillary duties. The value of this role for logistical support should not be underestimated by funding agencies.

### Agree to Flexibility for Management Prioritization

Flexibility must be present if two‐way engagement at the research–management–policy nexus is to be of value. Importantly, funding institutions must agree with the idea of plasticity in the research direction if needed. With management input comes the realization that a project’s initial direction may not be the highest priority need or provide for product buy‐in or implementation. Thus, researchers must have the ability to discuss shifts (not an entire rescoping) in direction or intended products with the funding organization, and a process needs to be in place to allow for these considerations. Including a representative from the funding agency on the MAB can facilitate these conversations to avoid the principal investigator (i.e., grant recipient) engaged in mediating between the MAB and the funding agency. Instead, the funding agency’s representative will learn the agencies’ research needs from the MAB. For example, at the initiation of our project the threat of Asian carp invading the Great Lakes did not seem to be imminent, but during the project we identified the need to undertake a bio‐economic assessment of potential impacts to inform response actions and investment.

### Support for Sustaining the Relationships Through the Entire Process

At the outset of the project, agency staff must commit to the process and engagement in order to support successful outcomes for the project. Early in the project initiation, a process should be developed to periodically review the MAB membership for updates due to staff turnover or changes in project directions or scope. New participants may need to be added in order to ensure the MAB retains good representation and relevance for the research areas.

Processes should be established to sustain communication between meetings. Information should be shared prior to meetings to prompt interest and participation, but not overwhelm recipients with more detail than necessary. Similarly, post‐meeting communication should follow up on necessary topics raised in the meetings. Communication should be concise, timely, and have appropriate frequency. All participants must agree on what types of communication are mutually useful.

The duration of the project is important in terms of developing the relationships and trust needed for open dialogue. It will likely take one or two meetings before participants feel comfortable sharing their feedback, comments, and questions openly. Therefore, depending on the frequency of meetings, communication, or other interactions, 2–3 years is likely the minimum amount of time needed for a project of this type. Researchers and MAB members must, of course, pay attention to the funding expiration horizon throughout the project and manage time and resources so project objectives are reached before resources are expended. Attention should also be paid to policy timelines and imperatives so that opportunities to contribute to policy decisions are not missed.

### Determine an Endpoint for the Process

For the MAB to succeed there needs to be an achievable endpoint that defines success and a logical conclusion to the process. For the MAB members, management priorities evolve and new issues arise, competing with commitments to the MAB. Unless MAB participants can see progress towards an agreed‐upon outcome, continued participation becomes difficult to justify to their agency. In our case study, the project had a defined funding end point and participation was sustained because progress was observed informally through research outputs that resulted in uptake and measurable management actions (Table 1). Progress towards an agreed endpoint could also be formally measured against defined milestones that both assess progress and inform shifts in research direction or priorities.

At the conclusion of the process, the MAB and research team need to determine if there are outstanding needs and establish mechanisms for knowledge transfer to ensure information and subject experts are available to facilitate management. Often the onus for information transfer needs to pass to the management members of the MAB, so they become the agents of change among their management peers. Celebrate the conclusion of the project and define what happens at the end. If applicable, determine when additional funding will be sought and who will seek it to continue the project’s direction.

## CONCLUSION

Explicitly designing a process to co‐produce management‐relevant knowledge and facilitate the transfer of scientific outputs to management and policy resulted in outcomes that would not have occurred with the traditional research model, wherein researchers conduct research and managers sometimes learn the results. The approach we describe here would ideally be required for large multi‐collaborator grants awarded with the goal of producing research that is meaningful to management and policy institutions. To be successful, granting programs must continue to recognize the value of research transfer through co‐production efforts like that outlined here, support the increased time to completion that it may entail, accept adaptation or realignment of research questions during the project, and be willing to support additional funding for travel, administration, and communications. Key leaders and personalities need to match the roles in the process; great scientists may not be the best communicators and all managers may not be innovators. It is important to identify and define group leaders who can facilitate the transfer of information in both directions and utilize their strengths to the benefit of the entire group.

The model we have described is not appropriate for every project, and this is not the only model for transdisciplinary research (Hallett et al. 2017; Wall et al. 2017). Nevertheless, we believe that it is a general model that deserves more widespread adoption and support by funding agencies interested in improving outcomes in the management and policy surrounding fisheries sustainability, water pollution, transitions to renewable energy, increasing agriculture sustainability, climate change, and other urgent challenges.
